# A pragmatic randomized controlled trial of multi-dose oral ondansetron for pediatric gastroenteritis (the DOSE-AGE study): statistical analysis plan

**DOI:** 10.1186/s13063-020-04651-1

**Published:** 2020-08-24

**Authors:** Anna Heath, Juan David Rios, Sarah Williamson-Urquhart, Petros Pechlivanoglou, Martin Offringa, Christopher McCabe, Gareth Hopkin, Amy C. Plint, Andrew Dixon, Darcy Beer, Serge Gouin, Gary Joubert, Terry P. Klassen, Stephen B. Freedman, on behalf of the PERC-KIDSCAN DOSE-AGE Study Group, Stephen Freedman, Sarah Williamson-Urquhart, Terry Klassen, Tannis Erickson, Rick Watts, Pam Marples, Anna Heath, David Rios, Petros Pechlivanoglou, Chris McCabe, Gareth Hopkin, Amy Plint, Tremaine Rowe, Gary Joubert, Leslie Boisvert, Serge Gouin, Marie-Christine Auclair, Andrew Dixon, Manasi Rajagopal, Mithra Sivakumar, Darcy Beer, Jeannine Schellenberg

**Affiliations:** 1 University of Toronto, Toronto, Ontario Canada; 2 University College London, London, United Kingdom; 3 Child Health Evaluative Sciences, The Hospital for Sick Children, Toronto, Ontario Canada; 4 Pediatric Emergency Research Team, Alberta Children’s Hospital, University of Calgary, Calgary, Alberta Canada; 5 Institute of Health Policy, Management and Evaluation, University of Toronto, Toronto, Ontario Canada; 6 Division of Neonatology, The Hospital for Sick Children, University of Toronto, Toronto, Ontario Canada; 7 Institute of Health Economics, Edmonton, Alberta Canada; 8 Division of Emergency Medicine, Children’s Hospital of Eastern Ontario, Ottawa, Canada; 9 University of Ottawa, Ottawa, Canada; 10 Children’s Hospital Research Institute, Ottawa, Canada; 11 Stollery Children’s Hospital, University of Alberta, Women’s and Children’s Health Research Institute, Edmonton, Canada; 12 Pediatrics/Pediatric Emergency Medicine, Department of Pediatrics and Child Health, Children’s Hospital Research Institute of Manitoba, Winnipeg, Manitoba Canada; 13 Université de Montréal, Montréal, Québec Canada; 14 CHU Sainte-Justine, Montréal, Québec Canada; 15 Children’s Hospital, Western University, London, Ontario Canada; 16 University of Manitoba, Winnipeg, Manitoba Canada; 17 Children’s Hospital Research Institute of Manitoba, Winnipeg, Manitoba Canada; 18 Sections of Pediatric Emergency Medicine and Gastroenterology, Department of Pediatrics, Alberta Children’s Hospital, Alberta Children’s Hospital Research Institute, Cumming School of Medicine, University of Calgary, Calgary, Alberta Canada

**Keywords:** Acute gastroenteritis, Emergency department, Pediatrics, Ondansetron, Statistical analysis plan

## Abstract

**Background:**

Acute gastroenteritis is a leading cause of emergency department visits and hospitalizations among children in North America. Oral-rehydration therapy is recommended for children with mild-to-moderate dehydration, but children who present with vomiting are frequently offered intravenous rehydration in the emergency department (ED). Recent studies have demonstrated that the anti-emetic ondansetron can reduce vomiting, intravenous rehydration, and hospitalization when administered in the ED to children with dehydration. However, there is little evidence of additional benefit from prescribing ondansetron beyond the initial ED dose. Moreover, repeat dosing may increase the frequency of diarrhea. Despite the lack of evidence and potential adverse side effects, many physicians across North America provide multiple doses of ondansetron to be taken following ED disposition. Thus, the Multi-Dose Oral Ondansetron for Pediatric Gastroenteritis (DOSE-AGE) trial will evaluate the effectiveness of prescribing multiple doses of ondansetron to treat acute gastroenteritis-associated vomiting. This article specifies the statistical analysis plan (SAP) for the DOSE-AGE trial and was submitted before the outcomes of the study were available for analysis.

**Methods/design:**

The DOSE-AGE study is a phase III, 6-center, placebo-controlled, double-blind, parallel design randomized controlled trial designed to determine whether participants who are prescribed multiple doses of oral ondansetron to administer, as needed, following their ED visit have a lower incidence of experiencing moderate-to-severe gastroenteritis, as measured by the Modified Vesikari Scale score, compared with a placebo. To assess safety, the DOSE-AGE trial will investigate the frequency and maximum number of diarrheal episodes following ED disposition, and the occurrence of palpitations, pre-syncope/syncope, chest pain, arrhythmias, and serious adverse events. For the secondary outcomes, the DOSE-AGE trial will investigate the individual elements of the Modified Vesikari Scale score and caregiver satisfaction with the therapy.

**Discussion:**

The DOSE-AGE trial will provide evidence on the effectiveness of multiple doses of oral ondansetron, taken as needed, following an initial ED dose in children with acute gastroenteritis-associated vomiting. The data from the DOSE-AGE trial will be analyzed using this SAP. This will reduce the risk of producing data-driven results and bias in our reported outcomes. The DOSE-AGE study was registered on ClinicalTrials.gov on February 22, 2019.

**Trial registration:**

ClinicalTrials.gov NCT03851835. Registered on 22 February 2019.

## Background

In the USA, nearly 50 million episodes of acute gastroenteritis occur each year [1]. In addition, nearly 2 million infected children are brought for emergency department (ED) care each year [2]. Rehydration is the cornerstone of care for minimally dehydrated children with acute gastroenteritis. They are encouraged to consume their preferred solutions [3] with electrolyte maintenance solution being recommended for those with some dehydration [4]. For children who experience vomiting, the antiemetic agent ondansetron is used to facilitate rehydration [5], with around 87% of pediatric ED physicians using antiemetics in these settings [6].

A 2016 meta-analysis identified that a single dose of oral ondansetron reduces the use of intravenous rehydration and hospital admissions, compared to placebo, in children who present for ED care with gastroenteritis-associated vomiting [7]. However, no evidence currently exists to indicate that additional doses of oral ondansetron taken at home lead to improved outcomes; some evidence even suggests an increase in adverse events (AE) [8]. Despite the lack of studies evaluating at-home oral ondansetron administration, it is commonly prescribed at discharge in many EDs across North America [9]. Thus, we designed the DOSE-AGE study to evaluate the effectiveness and safety of multiple doses of ondansetron provided to children with acute gastroenteritis-associated vomiting, following an initial ED dose.

The DOSE-AGE study is a phase 3 study that will investigate the effect of prescribing multiple doses of oral ondansetron, administered at home, on the probability of experiencing moderate-to-severe gastroenteritis in the 7 days following trial enrollment, compared to placebo. The study will enroll children and youth who present to any of six participating EDs with acute gastroenteritis-associated vomiting who are prescribed an initial dose in the ED. The study protocol is available [10] and this article outlines the statistical analysis plan (SAP) for the DOSE-AGE study. The analyses identified in this SAP will be included in future study abstracts and manuscripts. This SAP has been published before completing the data collection for the DOSE-AGE study and aims to reduce the potential for bias in the final trial report.

### Objectives

The primary aim of the DOSE-AGE study is to determine if the provision of a 6-dose (i.e., 2 days) supply of oral ondansetron prior to ED discharge will result in a reduction in the proportion of children who experience moderate-to-severe gastroenteritis symptoms, quantified by the Modified Vesikari Scale (MVS) score [11, 12], following discharge, compared to the placebo. We will enroll children and youth 6 months to 18 years of age, who were previously healthy, and present to one of six Canadian participating pediatric EDs with gastroenteritis-associated vomiting. The secondary objectives of the DOSE-AGE study are to investigate the effect of multi-dose oral ondansetron on possible adverse events (e.g., diarrhea), caregiver satisfaction, intravenous rehydration, and unscheduled health care provider visits.

## Methods/design

### Design and setting

The DOSE-AGE trial is a phase 3, placebo-controlled, double-blind, parallel design randomized controlled trial being conducted in 6 Canadian tertiary care pediatric EDs. All eligible participants will be administered a dose of ondansetron in the ED as part of their routine clinical care. The active treatment, to be compared with a placebo control, will be an at-home kit that contains 6 ondansetron elixir treatment doses plus 2 extra in case of spillage or vomiting, dosed at 0.15 mg/kg to a maximum single dose of 8 mg, to be administered at least 8 h after the initial clinical dose in the ED. Over the following 48 h, the intervention will be administered, at the discretion of the participant or caregiver, every 8 h with a maximum of 3 doses in a 24-h period. This “active” at-home treatment will be contrasted with the “placebo” treatment by randomizing participants in a 1:1 ratio to either the active or placebo treatment. All study personnel including data analysts will be blinded to the study group assignment. Participants will be followed up for 7 days after randomization with questionnaires issued 24 h, 48 h, and 7 days (i.e., 168 h) post-enrollment.

### Study protocol development and conduct

The DOSE-AGE study was registered on ClinicalTrials.gov on February 22, 2019 with the trial registration number NCT03851835. Research Ethics Board (REB) at each participating institution approved the study prior to commencing local enrollment. Informed consent will be obtained from caregivers and mature minors, as defined by local regulations, before randomization, data collection, or the performance of any study procedures. Assent will be obtained based on the child’s age, as appropriate. All consent and assent procedures will follow institutional REB guidelines. The DOSE-AGE study is part of the KidsCAN-PERC iPCT network [13]. This Canadian trials network is undertaking four trials using a centralized infrastructure for data management and trial oversight. Within this network, an independent data and safety monitoring board (DSMB) has been recruited to oversee the DOSE-AGE study.

### Randomization and data collection

Participants eligible for the DOSE-AGE study will be randomized in a 1:1 allocation ratio using a permuted block randomization procedure with various block sizes. We will stratify participants by clinical site and participant weight, < 20 kg and ≥ 20 kg, to ensure that site or weight-specific variations are distributed across treatment arms. We use weight, as opposed to age, to stratify participants as dosing is weight-based. Randomization will be undertaken using an Internet-based randomization service (www.randomize.net) to provide randomization kit numbers. A secure list will be sent to each site pharmacy where pharmacy staff will prepare consecutively numbered study kits according to the randomization schedule. The kits will then be grouped, and the assigned kit will be chosen at random within that group at the point of participant randomization.

All study participants data will be stored at the data coordination center (DCC) located at the Women and Children’s Health Research Institute (WCHRI) at the University of Alberta [14]. Data for analysis will be entered and stored in the REDCap electronic data capture system [15]. During the data collection process, some data may be recorded on paper and transcribed into the database or obtained from the participants and entered directly into the database. This electronic system will be based at the DCC and housed in a secure data center at the University of Alberta Hospital. Prior to analysis, all identifying information (e.g. contact information) will be removed and individual participants will be identified with a unique study identification number.

### Primary outcome

The primary outcome is the development of moderate-to-severe gastroenteritis in the 7 days following presentation to an ED. Moderate-to-severe gastroenteritis is defined as an MVS score in the 7 days post-randomization greater than or equal to 9 [12]. The MVS is a composite measure that includes seven components that are each assigned a score based on data collected between randomization and the completion of day 7 follow-up. The components will be calculated based on:

1. The duration of diarrhea following randomization, measured in hours.
2. The maximal number of episodes of diarrhea in any given 24-h period post-randomization.
3. The duration of vomiting following randomization, measured in hours.
4. The maximal number of episodes of vomiting in any given 24-h period post-randomization.
5. The maximal participant temperature recorded post-randomization.
6. The presence and type of unscheduled visits to a healthcare provider. If the participant records an ED visit to a participating site, this will be verified by database/chart review.
7. The type of treatment, if any, received for gastroenteritis in the 7 days post-randomization.

The scoring procedure for the MVS based on this information is given in Table 1.

**Table 1:** Modified Vesikari Scale scoring table. The scoring table to compute the Modified Vesikari Scale score from the survey outcomes in the DOSE-AGE trial

Scale component	Score on the Vesikari Scale			
	0 points	1 point	2 points	3 points
Duration of diarrhea symptoms (hrs)	0	< 96.0	96.0 to < 120	≥ 120
Maximum no. of watery stools in 24 h, across the 7-day follow-up	0	1–3	4–5	≥ 6
Duration of vomiting (hrs)	0	< 24.0	24.0 to < 48.0	≥ 48.0
Maximum no. of vomiting episodes in 24 h, across the 7-day follow-up	0	1	2–4	≥ 5
Maximum recorded rectal temperature (corrected) (°C)	< 37.0	37.0 to < 38.5	38.5 to < 39.0	≥ 39.0
Unscheduled health care visit	0	NA	Primary care	Emergency department
Treatment	None	Rehydration with intravenous fluids	Hospitalization	NA

Temperatures will be adjusted for the location of measurement: 1.1 °C will be added to axillary temperatures and 0.6 °C will be added to oral temperatures [16]

*hrs* hours, *No.* number, *IV* intravenous

### Secondary efficacy outcomes

The DOSE-AGE study will include six secondary outcomes to investigate the efficacy of multiple dose ondansetron compared to placebo.

1. The total number of episodes of vomiting across the 7 days post-randomization.
2. The time interval from randomization until the participant ceases vomiting. A participant with a 24-h period without either vomiting or diarrhea will be assumed to have ceased vomiting.
3. The proportion of participants who experience vomiting within 7 days of enrollment.
4. The proportion of participants who have an unscheduled health care provider visit within 7 days of enrollment.
5. The proportion of participants who receive intravenous rehydration within 7 days of enrollment.
6. Caregiver satisfaction with the treatment provided in the 48 h following ED disposition, as measured by a 5-point Likert scale.

### Safety outcomes

To investigate the safety of multiple doses of ondansetron, we will record the total number of diarrheal episodes during the 48-h following randomization, the maximal number of diarrheal episodes in a 24-h period during the 7 days following randomization, and the number of participants who experience any serious adverse events (SAEs), palpitations, pre-syncope/syncope, chest pain, and arrhythmias.

### Sample size calculation

The primary outcome in the DOSE-AGE trial is binary, the development of moderate-to-severe gastroenteritis in 7 days following randomization. Pilot data indicate that 30% of participants will have moderate-to-severe disease following the index visit [17, 18]. A survey of experts indicated that an absolute risk reduction of 10% would constitute a minimal clinically important difference (MCID) [19]. Therefore, our sample size calculation assumed a 30% event rate in the control group for which we desire to detect an absolute beneficial treatment effect of 10% with 90% power. Using a two-sided type I error of 0.05, the total sample size for the DOSE-AGE trial is 784 participants. We assumed a 10% loss to follow-up $\left(\frac{784}{0.9}=870\right)$, 5% drop out, and 3% drop in rate (i.e., caregivers who are provided with ondansetron outside the study protocol) $\left(\frac{870}{0.92^2}=1030\right)$. Thus, the total number randomized will be 1030. As a sensitivity analysis, we have computed the power for the DOSE-AGE trial based on potential alternative event rates for the active treatment and placebo groups (Table 2).

**Table 2:** Expected power for different event rates. The statistical power of the DOSE-AGE trial with 784 participants based on different assumptions for the true probability of moderate-to-severe gastroenteritis in 7 days following enrollment, as measured by the Modified Vesikari Scale score

Event rate for placebo control	Event rate for multi-dose ondansetron	Absolute risk reduction with multi-dose ondansetron	Statistical power to detect difference
**0.25**	**0.15**	**0.10**	**0.94**
**0.25**	**0.2**	**0.05**	**0.39**
**0.25**	**0.25**	**0.0**	**NA**
**0.3**	**0.15**	**0.15**	**0.999**
**0.3**	**0.25**	**0.05**	**0.35**
**0.35**	**0.15**	**0.2**	**~ 1**
**0.35**	**0.2**	**0.15**	**0.997**
**0.35**	**0.25**	**0.1**	**0.87**

### Interim analysis and stopping guidance

There are no planned interim analyses for the primary or secondary outcomes and early stopping for efficacy or futility will not be considered due to concerns about over- or under-estimating effects [20, 21]. The DSMB will consider stopping for safety. Statisticians tasked with providing the interim analyses to the DSMB will be blinded to the identity of the treatment arms, and DSMB reports will use treatments “A” and “B” throughout the reporting. The DSMB can be unblinded to treatment arm if required.

## Statistical analysis plan

### Statistical principals

Outcomes will be collected using follow-up surveys provided to caregivers 24 h, 48 h, and 168 h after randomization. Following ED disposition, study staff will contact caregivers using their preferred method of communication, e-mail or telephone, to collect the outcomes. The final analysis comparing oral ondansetron to placebo will take place in one stage with the main results prepared after every participant has completed the protocol and all data have been collected and cleaned, and the database has been locked. The analysis will be performed blinded to participant treatment allocation.

The final trial analysis will test for the superiority of the active treatment compared to placebo for all primary and secondary outcomes. We will use an intention-to-treat (ITT) principle and include all randomized participants, regardless of whether they adhered to the protocol. The safety analysis population will be any participants that took at least one dose of the study medication. We will declare statistical significance at the 5% level using two-sided tests for all inferential analyses. We will use the Bonferroni-Holm correction to maintain the family-wise error rate at 5% for the secondary outcomes, although we note that these analyses are exploratory as the trial has not been powered for these comparisons. We will report all estimates for the treatment effects using 95% confidence intervals, calculated using SAS or R [22, 23].

### Handling of missing data

Subjects who withdraw from the study or are lost to follow-up will have all available data used in the analysis. Data from the follow-up questionnaires, required to derive the primary outcome, may be missing. In these cases, we will use multiple imputation [24]. To minimize the use of imputation, only missing components will be imputed and then used to calculate variables, as required. For example, if the end date of diarrhea is missing, then this will be imputed and then used to calculate the duration of diarrhea. We aim to impute and analyze 10 datasets but will increase that number if the efficiency of the imputed datasets is less than 0.99 or if the proportion of missing observations is greater than 10% [25]. If greater than 10% of subjects are withdrawn or lost to follow-up, baseline characteristics and other information will be used to assess whether there is difference between the missing and non-missing subjects. If there is a difference, then we will explore the impact of alternative missing data assumptions on the results [26].

### Patient flow

We will use a CONSORT 2010 flow diagram to present patient flow for the DOSE-AGE trial. The diagram will report the number of participants deemed eligible for the trial at screening and those excluded as they met a study exclusion criteria. We will detail the number of participants who were randomized and received the randomized allocation.

Participants can withdraw from the DOSE-AGE study at any time and for any reason. The reason for voluntary withdrawal from the study will not be collected. Participants who choose to withdraw from the study can choose to discontinue the study intervention but complete the follow-up questionnaires or to withdraw from all further data collection. The site investigator may discontinue or withdraw a participant from the study if the participant meets an exclusion criterion that precludes study participation or if any clinical adverse event or other medical condition occurs such that study participation is not in the best interest of the patient. Reasons for withdrawal (i.e., caregiver- or investigator-initiated) and the number of participants lost to follow-up will be summarized by treatment arm.

### Protocol deviations

A minimum level of study doses (i.e., compliance) will not be required for this trial as it is not mandatory for caregivers to provide the study intervention to the participant. Thus, no deviation will be recorded if a caregiver does not administer the study intervention. Descriptive statistics on the number of doses provided will be presented by treatment group. Protocol violations are defined in keeping with ICH GCP guidelines.

The number and percentage of participants with protocol violations will be summarized by treatment group with details of the violation provided. Participants included in the ITT analysis data set will be used as the denominator to calculate protocol violation percentages. No formal testing will be undertaken.

### Baseline characteristics

All trial participants will be described in terms of age, sex, weight, whether they attend day care or school, and whether they have access to a primary care physician. Initial gastroenteritis severity will be recorded using vomiting and diarrhea duration and maximal 24-h frequency before attending the ED, the presence and height of fever, prior ED visits for the current illness, IV rehydration, hospitalization, baseline MVS, antibiotic usage in the preceding 14 days, dehydration assessment, and the need for hospitalization at the initial ED visit.

Our presentation of these baseline characteristics will depend on the type of data. In particular, categorical variables will be compared between the active and placebo groups using frequency counts and percentages. All continuous variables will be presented using the mean, median, standard deviation, and interquartile range. Baseline characteristics will not be subject to formal statistical testing.

### Analysis for the primary endpoint

The primary outcome, occurrence of moderate-to-severe gastroenteritis, will be compared across treatment groups using the logistic mixed model, adjusted for site and weight category. We will provide summary statistics of the effect size by reporting the absolute risk difference, relative risk, and odds ratios across the two treatment groups, alongside 95% confidence intervals. Confidence intervals will be obtained using the profile likelihood method with identity, log, and logit link functions, respectively, in a generalized linear mixed model [27]. If any participants are randomized within an incorrect stratum due to misspecification at time of randomization, the actual rather than assigned category will be used in the primary analysis.

### Analysis for secondary endpoints

#### Secondary outcomes

Secondary outcome 1 records the number of episodes of vomiting within the 7-day study follow-up. This will be compared across treatment groups using a generalized linear model to compare differences between Poisson rates while adjusting for stratification by site and weight. Secondary outcome 2 will assess differences in the duration of vomiting using the Van Elteren test, stratified by site and weight. If greater than 10% of participants still experience symptoms at the 7-day follow-up questionnaire, we will use a suitable procedure to take account of censoring.

Secondary outcomes 3–5 are binary and will be compared across treatment groups using logistic mixed models, stratified by site and weight. We will report the appropriate summary statistics for all these analyses with the corresponding adjusted 95% confidence intervals. Finally, we will test for differences in caregiver satisfaction (secondary outcome 6) using a Mann-Whitney *U* test.

#### Safety outcomes

We will undertake formal statistical testing for the frequency and maximum number of diarrhea episodes during the 7-day follow-up period. To test for differences in the frequency, we will use a generalized linear model to compare differences between Poisson rates while adjusting for stratification. We will then use a Mann-Whitney *U* test to determine whether there are differences in the maximum number of diarrhea episodes in 24 h. All other safety outcomes will be reported using frequencies.

#### Subgroup analyses

We will consider four pre-specified subgroup analyses of the primary outcome based on:

1. Sex
2. Age—6 months to < 3.0 years, 3.0 to < 6.0 years, 6.0 to < 10.0 years, and ≥ 10.0 years
3. Vomiting frequency, > 10 episodes in preceding 24 h
4. Presence of diarrhea (yes/no) in preceding 24 h

We will declare a significant subgroup effect if the interaction between assigned treatment and the subgroup factor is significant in the appropriate statistical model at a significance level of 0.05/4 = 0.0125. Logistic regression models will be used with a main effect for treatment, a main effect for the subgroup variable of interest, and an interaction between the subgroup variable of interest and the treatment. We will also stratify by site and weight in this subgroup analyses. A subgroup analysis for safety outcomes will also be undertaken by only including participants who received 3 or more doses of the study medication to assess the impact of multiple doses of ondansetron on diarrhea and other safety outcomes.

#### Additional analyses

To estimate the individual-specific rather than the population level effects, we will perform secondary analyses of the MVS score outcomes by assessing the treatment effect after adjustment for covariates, including:

- Baseline MVS (actual score)
- Duration of symptoms prior to enrollment (< 48 h vs. 48 h or more)
- Age
- Sex
- Study site
- Antibiotic usage during the 14 days prior to the ED visit
- Severity (i.e., frequency) of baseline diarrhea and vomiting
- Dehydration assessment
- Need for hospitalization at the index visit

We will use a logistic regression model employing the presence of moderate-to-severe disease as the dependent variable. We will also analyze the MVS outcome as a continuous variable using a linear mixed model, if appropriate, or a Van Elteren test, stratified by center and weight, to compare across treatment groups.

We will additionally analyze the total number of days that participants experience vomiting (i.e., secondary outcome 1) using a linear regression model to adjust for possible effects of the above covariates. We will also undertake a Bayesian exploratory analysis for the primary and secondary outcomes [28]. Finally, the DOSE-AGE trial is accompanied by a discrete choice experiment (DCE) aimed at examining caregiver preferences for the components of the MVS score. We will use the DCE to develop a preference-weighted scoring system for the MVS score. As an additional analysis, we will undertake the primary analysis using this preference-weighted MVS score as a continuous variable.

### Trial status

The DOSE-AGE study was registered on February 22, 2019, and started recruitment in September 2019 at the Alberta Children’s Hospital. The final institution is expected to begin recruitment of patients by February 2020. Recruitment is currently underway and is expected to complete around June 2022. The database will be cleaned and checked for completeness before the data are analyzed. At this point, the database will be locked, and the statistical analysis will be undertaken using the methods specified in this SAP.

## Data Availability

No datasets were used to develop this article as analysis was not undertaken. Thus, this consideration is not applicable.

## Abbreviations

AGE: Acute gastroenteritis
DSMB: Data Safety and Monitoring Board
MVS: Modified Vesikari Score
ED: Emergency department
DOSE-AGE: Multi-DOSE Oral Ondansetron for Pediatric Acute Gastroenteritis
(S)AE: (Serious) adverse events
CONSORT: Consolidated Standards of Reporting Trials
IV: Intravenous
ITT: Intention-to-treat
DCC: Data coordination center
WCHRI: Women and Children’s Health Research Institute
